# Effects of intradialytic resistance exercise on systemic inflammation in maintenance hemodialysis patients with sarcopenia: a randomized controlled trial

**DOI:** 10.1007/s11255-019-02200-7

**Published:** 2019-07-03

**Authors:** Zhi-Juan Dong, Hai-Lin Zhang, Li-Xia Yin

**Affiliations:** 1 Department of Nursing, The First People’s Hospital of Lianyungang, No. 182, Tongguan North Road, Haizhou District, Lianyungang, Jiangsu China; 2 Department of Hemopurification Center, The First People’s Hospital of Lianyungang, Lianyungang, Jiangsu China

**Keywords:** Intradialytic resistance exercise, Maintenance hemodialysis, Sarcopenia, Systemic inflammation

## Abstract

**Purpose:**

To investigate the effect of intradialytic resistance exercise on inflammation markers and sarcopenia indices in maintenance hemodialysis (MHD) patients with sarcopenia.

**Methods:**

Forty-one MHD patients with sarcopenia were divided into an intervention group (group E, *n* = 21) and a control group (group C, *n* = 20). Group C patients only received routine hemodialysis care, whereas group E patients received progressive intradialytic resistance exercise with high or moderate intensity for 12 weeks at three times per week (using the weight of the lower limbs and elastic ball movement of the upper limb) on the basis of routine hemodialysis care.

**Results:**

After 12 weeks, a significant difference in physical activity status (maximum grip strength, daily pace, and physical activity level), Kt/V, and C-reactive protein was found between groups E and C. Inflammatory factors (interleukin (IL)-6, IL-10, and tumor necrosis factor(TNF)-α) increased or decreased more significantly in group E than in group C.

**Conclusions:**

This study showed that intradialytic resistance exercise can improve physical activity effectively and reduce microinflammatory reactions even if this simple exercise does not affect the muscle mass in MHD patients with sarcopenia.

## Introduction

Sarcopenia, a newly recognized geriatric syndrome [1], with a prevalence of 4.8–31.0% has seriously affected the daily lives of people with chronic diseases [2, 3]. Disease characteristics are long-term muscle protein imbalance and limited physical activity. Moreover, the amount of protein in the human body accounts for about 20% of the total muscle. As age advances, the protein intake is relatively insufficient, resulting in protein synthesis, metabolism reduction, and weight loss. In addition, many clinical developments of chronic diseases and malignant tumors occur. These conditions used up protein to varying degrees. Generally, functions of the human heart and lungs gradually decrease with age, and muscle mass gradually shrinks, resulting in limited physical activity, further lack of physical activity, and ultimately occurrence of sarcopenia [4, 5]. The maintenance hemodialysis (MHD) patients as the representative of the sedentary group [6] have a sarcopenia prevalence of 13.7–73.5% [7, 8]. The situation is grim and needs to be strengthened as sarcopenia is far more prevalent in the ordinary elderly population.

Hemodialysis (HD) provides increased opportunities for endotoxin influx, recurrent infections, and immune activation leading to chronic systemic inflammation [9]. End-stage renal disease (ESRD) patients consequently have a dysfunctional immune system that is both chronically overactivated and anergic [10, 11]. In addition, renal function in patients with ESRD is almost or completely lost, and as the disease worsens, symptoms associated with sarcopenia such as muscle atrophy, decreased muscle strength, and decreased muscle function gradually appear [12, 13]. The low exercise capacity caused by muscle atrophy is an independent predictor of death in ESRD patients. Both chronic inflammatory state and physical inactivity are crucial in the pathogenesis of sarcopenia that is very common in MHD patients. Physical inactivity is a major contributing factor to chronic inflammation and protein energy wasting [14]. Consequently, physical inactivity and chronic inflammatory state can form a vicious cycle in MHD patients with sarcopenia.

Classic interventions to counteract age-related muscle wasting mainly focus on resistance training and/or protein supplementation to overcome anabolic inflexibility from which elderly suffer [15–17]. Although the elderly benefit from these classic interventions, the therapeutic potential of anti-inflammatory strategies is of great interest, as these resistance exercises might add up to the anabolic effect of protein supplementation. Some reports have confirmed the safety and effectiveness of exercise in MHD patients [18–20]. However, no intradialytic exercise is established for MHD patients with sarcopenia. Therefore, in this study, intradialytic resistance exercises were carried out in MHD patients with sarcopenia to investigate whether physical exercise can improve systemic inflammation and sarcopenia indices in such population.

## Materials and methods

### Subjects

The study population was composed of 45 prospectively recruited, voluntary MHD patients with sarcopenia. Prior to enrollment, a total of 341 MHD patients who were treated in the Hemopurification Center, The First People’s Hospital of Lianyungang, from May 2017 to July 2017, were selected for the detection of sarcopenia, but only 45 MHD patients with sarcopenia were identified as study subjects. The Institutional Ethics Committee of The First People’s Hospital, Lianyungang, Jiangsu, approved the study protocol (2017–2019). Written informed consent was obtained from each participant.

The inclusion criteria were as follows: (1) 18–80 years old, (2) stable dialysis time ≥ 3 months; (3) no central system disease; (4) can walk independently, no physical disability, muscle strength ≥ III; (5) dialysis patients with upper limb internal hemorrhoids; (6) can communicate normally; and (7) voluntarily participate in this study. The exclusion criteria were as follows: (1) pregnant woman; (2) 3 months of bleeding or infection records; (3) cannot perform bioelectrical impedance analysis (BIA) test, such as cardiovascular stent implantation, pacemaker installation, artificial joint replacement or amputation surgery; (4) had other serious complications such as heart failure, serious infection, malignant tumors, etc.; and (5) patients with cognitive impairment and mental illness.

### Study design

**Fig. 1:**
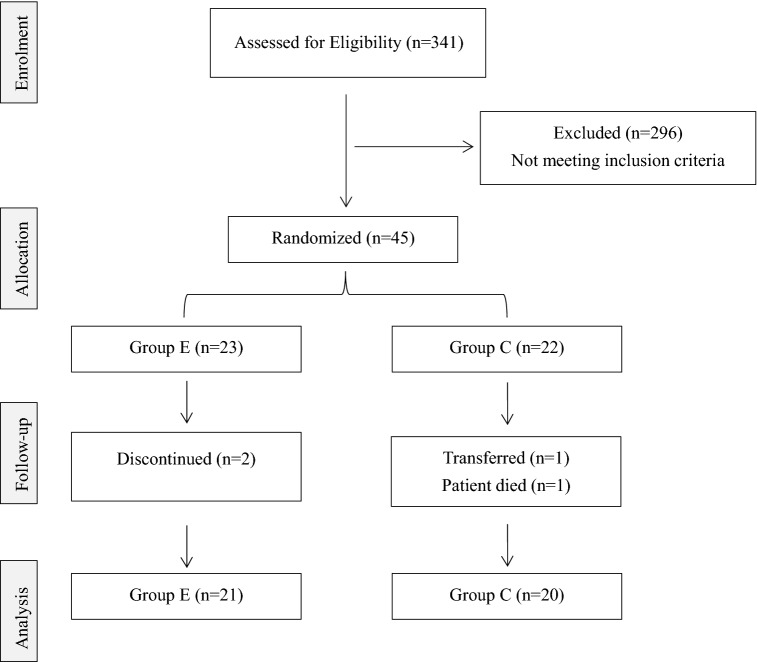
Consort flow chart for study participation

Subjects were divided into the intervention group (group E, *n* = 23) and control group (group C, *n* = 22) by the simple randomized method using a random number table (Fig. 1). A study has shown [21] that resistance exercise is the active movement of muscles to overcome external resistance. Compared with aerobic exercise, it focuses on improving cardiopulmonary function. Resistance exercise can improve muscle mass, muscle strength, and muscle function which appropriately target the symptoms of sarcopenia (reduced muscle cross-sectional area, muscle strength, and decreased systolic function [1]). Therefore, only patients in group C received routine HD care. Patients in group E performed progressive intradialytic resistance exercise with high or moderate intensity for 12 weeks at three times per week (using their own body weight and elastic balls) on the basis of routine HD care.

In the first week, the ankle weight was 0 kg, and quadriceps training board was used to assist the patient in low-intensity resistance training. According to the patient’s tolerance, the ankle weight of + 0.5 kg (single foot) per week until it was + 5 kg (one foot), with the angle of the training board reduced gradually (150°–90°) until it was removed. In the meantime, the untreated hand was holding the elastic ball for 10 × 10 performing each step of the upper limb resistance exercise. During the exercise, patients performed a 5-min warm up followed by a 1–2 h bout of intradialytic resistance exercise: for the one-leg-raise-and-down exercise, and upper limb bouncing ball movement which exerted pressure on the elastic ball and maximally maintained for 3–5 s for one cycle and then release it, both complete 10 × 10 cycles repeatedly.

During the exercise, a continuous electrocardiography monitor was used to monitor patient’s vital signs (blood pressure, heart rate, pulse, oxygen saturation, etc.) and observe the patient’s condition. Exercise should be stopped immediately when any of the following occurs [22]: low blood pressure or high blood pressure, over 80% of the maximum heart rate (maximum heart rate = 220 − age), Borg score > 15 points [23], hypoglycemia, dizziness, headache, pallor, chest pain, and exercise-induced breathing difficulties, etc.

### Diagnosis of sarcopenia and related symptoms

The diagnosis of sarcopenia mainly includes declining levels of muscle mass, muscle strength, and physical performance (simultaneously present low levels of muscle mass and muscle strength/muscle function). This study uses the Asia Working Group for Sarcopenia (AWGS) [1] to recommend a diagnostic threshold: BIA skeletal muscle mass index (SMI) (male < 7.0 kg/m^2^, female < 5.7 kg/m^2^), hand grip strength (HGS) (male < 26 kg, female < 18 kg), and daily gait speed (< 0.8 m/s).

On the dialysis day, HGS was measured before the dialysis procedure (the patient stood straight with the limbs naturally sagged [24], three measurements were attempted using an electronic dynamometer (EH101, Xiangshan, China), each time for 5 s, taking the maximum) and daily pace [25] (the patient walked on the ground, the time spent every 6 m of walking was recorded) using BIA technology [26, 27] (InBody770, InBody, China) to measure the body component-related nutritional indicators (body mass index (BMI), SMI, skeletal muscle mass (SMM), fat-free body weight (FFM), fat-free body mass (FFMI), waist-to-hip ratio (WHR), fat mass (FM), fat mass index (FMI)) within 30–60 min after dialysis on the same day.

### Physical activity status

In this study, we used the international physical activity questionnaire to assess the level of human activity effectively. The questionnaire mainly consists of four parts. According to the evaluation of patient’s sitting, walking, cycling, and running ability, weight, etc., patient’s physical activity level (light, moderate, and intense) is evaluated: the lower the level, the less the physical activity [28].

### Cytokine, albumin, and high-sensitivity C-reactive protein (hs-CRP) measurements

For the inflammatory modulation analyzes, 8 mL of venous blood was collected from the antecubital vein into tubes without anticoagulant in pre-HD, immediately after the HD session for both groups (post HD). Serum samples were separated by centrifugation for 5 min at 1048 g (2500 rpm), divided into aliquots, and frozen at − 80 °C for further analysis.

Serum IL-6, IL-10, and TNF-α were measured using enzyme-linked immunosorbent assays. hs-CRP was measured using particle-enhanced immunoturbidimetric assay. Serum albumin was measured using a colorimetric assay.

### Statistical analysis

Statistical analysis was performed using SPSS 23.0 (SPSS Inc., Chicago, IL, USA). Normal measurement data were expressed as mean ± standard deviation, and *t* test was used for comparison between groups. Non-normal measurement data were expressed by median and quartile, and nonparametric test was used for comparison between groups. The count data usage rate (%) is expressed as a comparison between groups using the *χ*^2^ test or Fisher’s exact probability method. Statistical significance of variables was established at the level *P* < 0.05.

## Results

In this study, all questionnaires (group E, *n* = 21; group C, *n* = 20) were returned, and the effectivity rate was 100%. There were no significant differences in the demographic characteristics and disease-related data of the study subjects (Table 1). There were also no differences in the major medical treatments, including antiaggregants, antihypertensive drugs, β-blockers, statins, and erythropoietin between groups C and E.

**Table 1:** Comparison of demographic characteristics and disease-related data of study subjects (*n* = 41)

	All subjects (%) *n* = 41	Group E (%) *n* = 21	Group C (%) *n* = 20	*x*^2^/*t*/*Z*/*F*	*P* 值
Age	60.0 (43.0, 68.0)	59.0 (32.5, 66.5)	62.5 (50.5, 70.0)	− 1.253	0.210
Sex				1.205	0.272
Male	21 (51.2)	9 (42.9)	12 (60.0)		
Female	20 (48.8)	12 (57.1)	8 (40.0)		
Dry weight (kg)	53.21 ± 9.36	52.03 ± 9.26	54.44 ± 9.55	− 0.817	0.419
Economic status				0.023	0.879
Feel financially difficult	20 (48.8)	10 (47.6)	10 (50.0)		
Feel not financially difficulty	21 (51.2)	11 (52.4)	10 (50.0)		
Marital status				0.067	0.796
Married	30 (73.2)	15 (71.4)	15 (75.0)		
No spouse	11 (26.8)	6 (28.6)	5 (25.0)		
Educational level				3.027	0.463
Elementary school and below	9 (22.0)	3 (14.3)	6 (30.0)		
Junior high school	3 (7.3)	2 (9.5)	1 (5.0)		
High school and secondary School	23 (56.1)	14 (66.7)	9 (45.0)		
College and university	6 (14.6)	2 (9.5)	4 (20.0)		
Dialysis age	59.0 (31.5, 86.0)	69.0 (31.5, 87.5)	57.5 (32.5, 86.5)	− 0.157	0.876
Primary disease				2.289	0.512
Chronic nephritis	21 (51.2)	9 (42.9)	12 (60.0)		
Diabetic nephritis	6 (14.6)	3 (14.3)	3 (15.0)		
Hypertensive nephritis	5 (12.2)	4 (19.0)	1 (5.0)		
Others	9 (22.0)	5 (23.8)	4 (20.0)		
Complication				0.196	0.658
Yes	15 (36.6)	7 (33.3)	8 (40.0)		
No	26 (63.4)	14 (66.7)	12 (60.0)		

### Changes in physical activity and biochemistry data

There was no significant difference in physical activity and biochemistry data between groups E and C before the intervention (*P* > 0.05). After the 12-week intervention period, the differences were statistically significant in maximal grip strength, daily stride rate, and physical activity level between groups E and C (*P* < 0.05). There was no statistically significant difference in neutrophil ratio, hemoglobin, serum creatinine, normalized protein catabolic rate, and subjective global assessment, but there was a statistically significant difference in the patient’s Kt/V and nutritional index serum albumin (Table 2).

**Table 2:** Comparison of physical activity status and biochemical data between groups E and C before and after intervention ($\bar{x} \pm s$)

	Group E (*n* = 21)		Group C (*n* = 20)		*t/F*	*P* 值
	Before	After	Before	After		
Maximum grip strength (kg)	22.23 ± 5.27	26.03 ± 3.85	20.99 ± 6.05	21.34 ± 6.16	2.937	0.006
Daily pace (m/s)	0.97 ± 0.35	1.16 ± 0.33	0.98 ± 0.25	0.94 ± 0.26	2.305	0.027
Physical activity level (*n*, %)					16.137^a^	< 0.01
Mild	12 (57.1)	3 (14.3)	15 (75.0)	13 (65.0)		
Moderate	6 (28.6)	6 (28.6)	5 (25.0)	6 (30.0)		
Severe	3 (14.3)	12 (57.1)	0 (0.0)	1 (5.0)		
Neutrophil ratio (%)	65.33 ± 10.30	66.39 ± 10.24	69.83 ± 6.55	71.83 ± 8.29	− 1.875	0.068
Hemoglobin (g/L)	107.10 ± 21.52	103.43 ± 18.03	105.95 ± 19.54	105.00 ± 20.27	− 0.262	0.795
Serum creatinine (mg/dL)	9.17 ± 2.23	9.62 ± 0.27	9.36 ± 2.83	9.60 ± 3.29	0.028	0.978
Kt/V	1.61 (1.46, 1.95)	1.70 ± 0.27	1.53 (1.45, 1.70)	1.47 ± 0.32	2.464	0.018
Serum albumin (g/L)	37.19 ± 3.72	39.84 ± 3.37	37.79 ± 2.68	36.01 ± 6.39	2.384	0.024
nPCR (g/kg/d)	1.39 ± 0.41	1.74 ± 0.30	1.38 ± 0.69	1.78 ± 0.49	− 0.321	0.750

*P* two groups after the intervention, *Kt/V* dialysis adequacy, *nPCR* standard protein decomposition rate

^a^*F* value

### Changes in muscle-related nutritional indicators

There were no significant differences in body component-related nutritional indicators (BMI, SMI, SMM, FFM, FFMI, WHR, FM, FMI) between groups E and C before intervention. Before the intervention, 4 weeks after the intervention, and 12 weeks after the intervention, there were no significant differences in the comparison of the same period between the groups E and C (Table 3). BMI and FMI differences were statistically significant only in the comparison between groups (Table 4).

**Table 3:** Comparison of BIA-related nutritional indicators at different time points before and after intervention in the two groups

	Group E (*n* = 21)			Group C (*n* = 20)		
	Before	4 weeks	12 weeks	Before	4 weeks	12 weeks
BMI	18.96 ± 3.08	18.83 ± 3.07	19.44 ± 3.26	20.49 ± 3.41	20.45 ± 3.25	20.82 ± 3.41
SMI	5.70 ± 0.80	5.54 ± 0.70	5.69 ± 0.84	5.87 ± 0.69	5.85 ± 0.69	5.90 ± 0.69
SMM (kg)	21.19 ± 3.65	20.60 ± 3.40	21.20 ± 3.93	21.06 ± 3.12	21.12 ± 2.97	21.21 ± 2.97
FM (kg)	10.10 (8.05, 18.50)	10.80 (8.90, 18.80)	10.00 (8.20, 20.55)	14.15 (9.13, 19.90)	14.35 (9.63, 19.90)	14.05 (11.55, 19.75)
FMI	3.40 (3.15, 6.85)	3.80 (3.30, 7.05)	3.90 (3.05, 7.45)	5.60 (3.28, 7.33)	5.60 (3.28, 7.18)	5.75 (4.32, 7.92)
FFM (kg)	39.66 ± 6.38	38.59 ± 5.91	39.77 ± 7.02	39.37 ± 5.24	39.05 ± 4.91	39.68 ± 4.96
FFMI	14.43 ± 1.71	14.09 ± 1.54	14.46 ± 1.88	14.78 ± 1.25	14.70 ± 1.31	14.89 ± 1.24
WHR	0.87 ± 0.06	0.90 ± 0.07	0.90 ± 0.07	0.91 ± 0.07	0.91 ± 0.07	0.91 ± 0.08

*BIA* bioimpedance analysis, *BMI* body mass index, *FM* fat mass, *FMI* fat mass index, *FFM* fat-free body weight, *FFMI* fat-free body mass, *SMI* skeletal muscle mass index, *SMM* skeletal muscle mass, *WHR* waist-to-hip ratio

**Table 4:** Repeated measures analysis of variance and simultaneous control analysis of BIA-related nutritional indicators in the two groups

	Repeated measurement data analysis of variance						Contrast analysis results			
	*F*_group_	*P*	*F*_time_	*P*	*F*_group × time_	*P*	*t/Z* ^a^	*P*	*t/Z* ^b^	*P*
BMI	5.301	0.027	0.310	0.705	0.017	0.974	− 1.637	0.110	− 1.321	0.194
SMI	1.807	0.187	0.346	0.676	0.122	0.855	− 1.390	0.172	− 0.887	0.381
SMM	0.032	0.858	0.162	0.831	0.146	0.845	− 0.527	0.601	− 0.009	0.993
FM	3.658	0.063	0.213	0.750	0.020	0.956	− 1.161^c^	0.246	− 1.461^c^	0.144
FMI	4.369	0.043	0.202	0.763	0.031	3.000	− 1.057^c^	0.291	− 1.383^c^	0.167
FFM	0.000	0.985	0.370	0.671	0.061	0.927	− 0.269	0.790	0.048	0.962
FFMI	1.990	0.166	0.488	0.593	0.106	0.877	− 1.367	0.179	− 0.874	0.387
WHR	2.017	0.163	0.123	0.851	0.136	0.839	− 0.569	0.573	− 0.798	0.430

*BIA* bioimpedance analysis, *BMI* body mass index, *FM* fat mass, *FMI* fat mass index, *FFM* fat-free body weight, *FFMI* fat-free body mass, *SMI* skeletal muscle mass index, *SMM* skeletal muscle mass, *WHR* waist-to-hip ratio

^a^Two groups after 4 weeks

^b^Two groups after 12 weeks

^c^*Z* value

### Changes in systemic inflammation

**Fig. 2:**
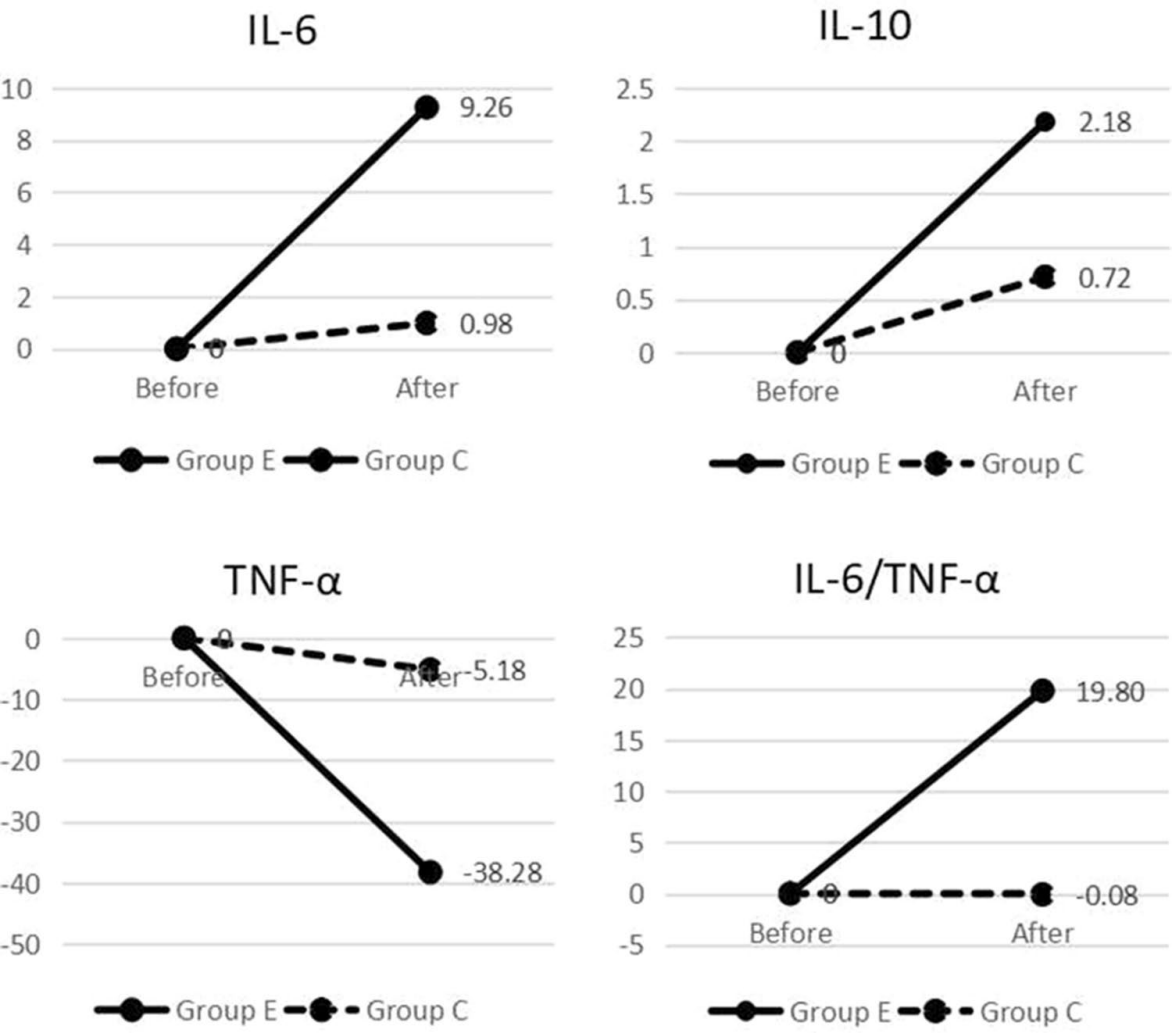
Comparison of the mean difference in inflammatory factors before and after intervention between groups E and C

There were no significant differences in CRP, IL-6, IL-10, TNF-α, IL-6/TNF-α between the two groups before intervention (*P* > 0.05). After the intervention, the difference in CRP between the two groups was statistically significant (*P* < 0.05) (Table 5). The mean values were compared between the two groups after the intervention in the course of the data between groups E and C (Table 6). IL-6 and IL-10 as representatives of anti-inflammatory factors were increased after the intervention and the pro-inflammatory factor TNF-α decreased significantly after the intervention (Fig. 2).

**Table 5:** Comparison of microinflammation status between the two groups

	Group E (*n* = 21)		Group C (*n* = 20)		*Z*	*P*
	Before	After	Before	After		
CRP (pg/mL)	2.23 (1.29, 5.24)	2.02 (1.40, 3.73)	2.91 (1.91, 8.48)	3.47 (1.95, 7.10)	− 2.335	0.020
IL-6 (pg/mL)	7.72 (4.95, 14.33)	9.43 (6.30, 12.47)	5.48 (3.89, 10.06)	7.93 (5.61, 11.31)	− 0.757	0.449
IL-10 (pg/mL)	0.82 (0.54, 0.94)	1.37 (1.18, 2.00)	0.79 (0.52, 1.43)	1.21 (0.93, 1.67)	− 0.939	0.348
TNF-α (pg/mL)	1.35 (0.59, 6.85)	1.26 (0.51, 8.96)	0.60 (0.20, 6.59)	4.45 (0.35, 12.19)	− 0.665	0.506
IL-6/TNF-α	5.83 (1.24, 13.30)	8.07 (1.91, 14.09)	7.73 (2.25, 17.85)	2.24 (0.64, 22.73)	− 1.174	0.241

*Z* and *P* are compared between the two groups after intervention

*CRP* C-reactive protein, *IL* interleukin, *TNF-α* tumor necrosis factor-alpha

**Table 6:** Comparison of the mean number of microinflammation factors between the two groups ($\bar{x}$)

	Group E (*n* = 21)		Group C (*n* = 20)		Difference between group E	Difference between group C
	Before	After	Before	After		
CRP (pg/mL)	3.71	2.79	5.86	5.24	− 0.92	− 0.62
IL-6 (pg/mL)	23.23	32.49	40.24	41.23	9.26	0.99
IL-10 (pg/mL)	1.22	3.4	3.83	4.55	2.18	0.72
TNF-α (pg/mL)	114.99	76.71	108.04	102.86	− 38.28	− 5.18
IL-6/TNF-α	10.59	30.39	10.45	10.37	19.8	− 0.08

CRP, C-reactive protein; IL, interleukin; TNF-α, tumor necrosis factor-alpha

## Discussion

The occurrence and development of sarcopenia in MHD patients have been paid attention at home and abroad. In this study, the prevalence of sarcopenia in MHD patients was 13.2%, of which the prevalence rate in 50-year-olds was 43.2%, and the prevalence rate in 60-year-olds was 55.4%, which is similar to the overall prevalence of sarcopenia in patients with MHD at home and abroad (13.7–73.5%) [7, 8].

This study focused on the effects of exercise on the dialysis process. Patients not only had high compliance (only two cases experienced falls), but also effectively improve systemic inflammation, dialysis adequacy, and physical activity, thereby improving the quality of life of MHD patients with sarcopenia.

### Improvement in systemic inflammation

MHD patients have been chronically inflamed. In the late 1990s, Zimm et al. [29] indicated that inflammation is closely related to cardiovascular disease, protein energy expenditure, and mortality in chronic kidney disease. In myasthenia, chronic inflammation affects muscle protein breakdown and synthesis through several signaling pathways, resulting in loss of muscle mass, muscle strength, and muscle function [30]. Greiwe et al. [31], based on the fact that muscle contraction can reduce systemic low-level inflammation, hypothesized that exercise can regulate inflammation, trigger hypothalamic–pituitary–adrenal axis and sympathetic nervous system, and stimulate the release of cortisol, adrenaline, and other hormones. A large number of studies have shown that [17, 32, 33] CRP is a typical manifestation of acute phase inflammation in human tissues, while IL-6 as an anti-inflammatory cytokine and is resistant to the pro-inflammatory cytokine TNF-α. It is known that excess adipose tissue increases the production of the pro-inflammatory cytokine TNF-α [32]. During exercise, the anti-inflammatory cytokine IL-6, by contracting skeletal muscle fibers, not only increases its production and release, but also stimulates other anti-inflammatory cytokines such as IL-10, and also inhibits the production of the pro-inflammatory cytokine TNF-α.

In this study, after the 12-week intervention period, the CRP level in the intervention group was significantly lower than that before the intervention, and the difference between the two groups was statistically significant. It is indicated that the anti-resistance movement during dialysis can reduce CRP. In addition, TNF-α in the intervention group was higher than before intervention and IL-6 and IL-10 decresed significantly when compared with that before intervention, consistent with the hypothesis on exercise and inflammation mentioned above. Similar to the results of Moraes et al. [33], there were no statistically significant differences in inflammatory cytokines. The possible causes are as follows: the microinflammation state is a chronic process in which short duration and moderate- to high-intensity events are associated with an overall reduction in circulating levels of inflammatory markers, but do not stimulate IL-6 release [34]; differences in laboratory testing methods used to measure different inflammatory markers; patient’s inflammatory cytokines were interfered with dialysis treatment. IL-6 and CRP increased significantly at 1–3 h after dialysis. After dialysis, IL-6 and CRP levels did not increase. TNF-α levels decreased significantly after dialysis but then returned to the baseline levels [35]; small sample size; and the variability of the muscle mass and endurance of the recruited participants.

### Positive impact on patient’s dialysis adequacy

Dialysis adequacy (Kt/V) is an important indicator of evaluating dialysis effectiveness which used a one-chamber urea kinetic model and reflects the ratio of the volume of urea removed in the dialysis to the total amount of water [36]. Whether MHD patients with dystrophy and dialysis treatment are inseparable, and dialysis treatment leads to waste of muscle protein, abnormal hormone synthesis, metabolic acidosis, etc. [37–40], are physiological and pathological factors for the development of myasthenia. Therefore, it is particularly important to improve the dialysis adequacy. During the dialysis, the movement can make the solute, such as urea in cells, advance into the blood circulation, so that the solute concentration gradient between the chambers is reduced, the imbalance is reduced, and the HD adequacy is further increased [41].

According to the Chinese Clinical Guidelines for Hemodialysis Adequacy (K/DOQI) (2015 Edition) [42], a single dialysis Kt/V ≥ 1.2 is recommended, and Kt/V ≥ 1.4 is better when conditions permit. In this study, the Kt/V of both groups was greater than 1.4, indicating that the dialysis effect of this research center is better. After 12 weeks of intervention, the difference of Kt/V between the two groups was statistically significant and indicated that resistance to dialysis can improve dialysis adequacy, consistent with Kirkman et al.’s report [43]. On the other hand, the percentage of improvement that occurred in dialysis adequacy was 5.6% after 12 weeks of intervention, and when compared it with aerobic exercise studies was 1.3% [14]. We can always prove that exercise can increase blood flow velocity in muscle tissue with high solute content such as uric acid, urea, and creatinine during the dialysis, thereby increasing the transport speed of solute in cells and a large amount of metabolic waste moves into the bloodstream, which helps promote HD. Adequacy can promote urea clearance and phosphate removal in the dialysate effectively.

### Improvement of the level of physical activity

Physical activity refers to various activities that result in a significant increase in energy expenditure due to skeletal muscle contraction [44]; individuals must have sufficient physical activity to meet daily needs. An international multicenter joint research has shown [45] that sedentary lifestyles in MHD patients result in generally lower levels of physical activity. Grip strength and daily pace are simple, widely used indicators of muscle strength assessment, and their baseline values are linearly related to daily physical activity [24, 46]. Studies by Isoyama et al. [47] reported reduced muscle strength compared with simple muscle mass reduction, which increased the risk of death in MHD patients. Regular resistance exercise is effective in improving muscle strength of these patients. In the study by Olvera et al. [48], MHD patients performed resistance exercise twice a week during dialysis treatment for a total of 12 weeks, and the grip strength increased from 19.6 to 21.2 kg. (*P* < 0.05), similar to the results of Song et al. [49]. In foreign studies, most of the 6-min walking test results replace the daily pace, as the two have the same meaning [25]. Frih et al. [30] divided the MHD patients into the intervention group and control group, in which the intervention group performed the exercise for 4 times a week at 4 months; after the intervention, the grip strength test (+ 23.54%) and 6-min walk test improved (+ 15.94%) in the intervention group. There was a statistically significant difference from the control group. The results in the present study are consistent with the results of the above studies. After the 12-week exercise intervention in the intervention group, the percentage of improvement occurred in maximum grip strength and daily pace was 18.9% and 19.6%, separately. This shows that the anti-resistance exercise improves the patient’s quality of life by directly involving the muscles, improving the muscle strength and self-care ability of the patients.

At the same time, the level of physical activity of the patients increased significantly after the 12-week intervention, and the proportion of mild physical activity levels decreased from 57.1 to 14.3%. However, there was no significant change in physical activity before and after the intervention in group C, indicating that the progressive intradialytic resistance exercise can improve the physical activity level of the patient and ultimately improve the quality of life. In this study, the reason might be that MHD patients were guided to progressive high-intensity dialysis exercise, making full use of the patient’s treatment time, which helped to improve patient’s participation during exercise. During the dialysis treatment, the medical staff and rehabilitation staff can monitor changes in patient’s condition at any time and adjust the exercise intensity according to the patient’s condition to ensure the safety of the exercise and at the same time achieve personalization of the exercise therapy.

### Comparison of BIA-related nutritional indicators

BIA is used commonly for the diagnosis of sarcopenia in clinical practice [50], and its nutritional indicators are highly analyzable. Diagnostic indicators for patients with sarcopenia include muscle mass, muscle strength, and/or physical function. While MHD patients generally suffer from low muscle strength due to prolonged sedentary reasons, the diagnosis of sarcopenia is driven by muscle mass in the MHD group [51]. The improvement of MHD with sarcopenia should be based on changes in muscle mass. A large number of research scholars have used randomized controlled trial to verify whether resistance exercise intervention can improve muscle mass. Kirkman et al. [52] followed 23 MHD patients to perform resistance exercise three times a week for a total of 12 weeks, resulting in a muscle volume of 2822–2906 cm^3^, which was statistically significant compared with the control group and similar to the results of Olvera et al. [48].

However, in this study, group E performed 12-week high-intensity resistance exercise at three times per week, and there was no statistical significance in the muscle quality-related indicators (SMI, SMM, FM, FMI, FFM, FFMI, WHR) between groups E and C (*P* > 0.05), but there were fluctuations in the values. Consistent with the conclusions of Connie et al. [53], simple resistance exercise does not increase muscle mass. This may be due to the following: participants have a high degree of variability, limited tools and equipment cannot capture patient’s physical changes over time, the synthesis and decomposition of muscle protein caused by exercise, short intervention time; and type II error caused by a small sample size of the experimental study.

## Conclusions

Intradialytic resistance exercise with medium to high intensity is a safe, low cost, and efficient approach to improving the muscle status and dialysis effect of MHD patients with sarcopenia. Meanwhile, we found simultaneous attenuation of inflammatory responses, which might contribute to these beneficial effects of exercise. Large-scale randomized controlled trials are required to optimize the intradialytic resistance exercise program for MHD patients with sarcopenia.
